# Babassu Mesocarp-Based Coating with Amazonian Plant Extracts Obtained Using Natural Deep Eutectic Solvents (NADES) for Cherry Tomato Preservation

**DOI:** 10.3390/foods15010074

**Published:** 2025-12-25

**Authors:** Carollyne Maragoni-Santos, Camila Marcolongo Gomes Cortat, Lilia Zago, Stanislau Bogusz Junior, Tatiana Castro Abreu Pinto, Jefferson Santos de Gois, Bianca Chieregato Maniglia, Ana Elizabeth Cavalcante Fai

**Affiliations:** 1 Food and Nutrition Graduate Program, Federal University of the State of Rio de Janeiro (UNIRIO), Rio de Janeiro 22290-240, RJ, Brazil; carolmaragoni@edu.unirio.br; 2 Laboratory of Multidisciplinary Practices for Sustainability (LAMPS), Department of Basic and Experimental Nutrition, Institute of Nutrition, Rio de Janeiro State University (UERJ), Rio de Janeiro 20550-900, RJ, Brazil; camilamgcortat@gmail.com (C.M.G.C.); lilia.zago@gmail.com (L.Z.); 3 São Carlos Institute of Chemistry (IQSC), University of São Paulo (USP), São Carlos 13566-590, SP, Brazil; stanislau@iqsc.usp.br (S.B.J.); biancamaniglia@iqsc.usp.br (B.C.M.); 4 Paulo de Goes Institute of Microbiology, Federal University of Rio de Janeiro (UFRJ), Rio de Janeiro 20550-013, RJ, Brazil; tcap@micro.ufrj.br; 5 Department of Analytical Chemistry, Rio de Janeiro State University (UERJ), Rio de Janeiro 20550-900, RJ, Brazil; jeffersonsgois@gmail.com

**Keywords:** NADES, Uxi-amarelo bark extract, Jambolan leaf extract, babassu mesocarp coating, active coating

## Abstract

Active biopolymer-based packaging incorporating phytochemicals offers promising sustainable alternatives for reducing postharvest losses and extending food shelf life. This study aimed to advance natural food packaging by (i) developing and characterizing natural deep eutectic solvents (NADES) using choline chloride combined with citric acid (CC-CA), glucose (CC-G), and urea (CC-U); (ii) obtaining bioactive extracts from Uxi bark and Jambolan leaves using these NADES; (iii) formulating babassu mesocarp-based coatings enriched with CC-CA extracts; and (iv) evaluating their application on cherry tomatoes. CC-U exhibited the lowest density (1.152 ± 0.037 g cm^−3^), while CC-G demonstrated the highest viscosity (18.375 ± 0.430 mPa s), and CC-CA presented the lowest polarity parameter (E_NR_) value (44.6 ± 0.1 kcal mol^−1^). Extracts obtained with CC-CA (YU-CA and JL-CA) showed high extraction efficiency, strong antioxidant activity (DPPH inhibition > 95%), and antimicrobial activity, particularly against *Pseudomonas aeruginosa*. Although the coatings exhibited lower bioactivity than the extracts, they effectively reduced weight loss, maintained firmness, and preserved the microbiological quality of tomatoes for up to 9 days. Sensory analysis of bruschetta prepared with coated tomatoes indicated high acceptance (>80%). Babassu mesocarp-based coatings enriched with Amazonian plant extracts emerge as an innovative active packaging strategy aligned with the 2030 Agenda.

## 1. Introduction

Petroleum-based plastics dominate food packaging due to their low cost and performance but raise serious ecological concerns due to their non-renewable origin and persistence in the environment [1,2]. With population growth and urbanization, it is becoming increasingly important to maintain food quality and safety, and packaging plays a key role in reducing post-harvest losses and waste, an issue of social and environmental urgency [3]. In this context, alternatives such as active coatings made from biobased compounds, including polysaccharides, proteins, and lipids, have gained increasing attention [1,4,5,6]. Babassu (*Attalea speciosa*) mesocarp, a by-product of oil extraction, after drying and milling, yields a flour rich in carbohydrate (84.57%, of which 56.40% is starch), proteins (1.8%), lipids (0.33%), and fibers (11%) [7]. Its composition makes it a valuable raw material for the development of biodegradable packaging. Ferreira et al. [8] developed coatings of cassava starch enriched with babassu flour and applied them to cagaita (*Eugenia dysenterica*) and mangaba (*Hancornia speciosa*) fruits. The coated fruits exhibited significantly lower weight loss than the uncoated ones and also showed a delayed color change.

Natural plant extracts rich in phenolic compounds are a promising substitute for synthetic additives, as they combine the efficacy of preservation with lower health risks and environmental impact [9]. The yellow uxi (*Endopleura uchi*), native to the Brazilian Amazon, is traditionally consumed in the form of infusions or extracts and contains bioactive compounds such as gallic acid, bergenin, isocoumarin, and maslinic acid, which have antioxidant, anti-inflammatory, antidiabetic, and antimicrobial effects [10,11,12]. Similarly, the leaves of Jambolan (*Syzygium cumini*) are rich in flavonoids (catechin and quercentin) and phenolic acids (gallic acid, caffeic acid, ferulic acid, ellagic, acid and p-coumaric acid) with strong antioxidant and antimicrobial potential [13,14]. Despite this bioactivity, both species are still under-researched for active packaging applications.

Natural deep eutectic solvents (NADES) are efficient, safe extraction media produced from natural metabolites such as urea, carboxylic acids, sugars, amino acids, choline chloride, and polyols [15,16,17,18]. NADES are formed by hydrogen bond acceptors and donors; are recyclable, thermally stable, and biocompatible; and act as plasticizers, which facilitates the direct incorporation of extracts into films and coatings [19,20,21]. Recent studies have shown that NADES-extracted polyphenols from purple cabbage, *Larrea divaricata*, *Rosa roxburghii leaves*, and purple sweet potato improve both the bioactivity and mechanical properties of biopolymer films [20,21,22,23]. The extraction of phytochemicals with NADES and their incorporation into active coatings is therefore a promising strategy to extend shelf life, reduce post-harvest losses, and maintain food quality. This is urgently needed as 30–40% of fruits and vegetables are lost after harvest [24]. Cherry tomatoes are an example of this vulnerability. Their thin skin and juicy flesh make them highly perishable [25]. It has already been shown that edible coatings enriched with bioactive compounds significantly extend their shelf life [26,27,28].

This study aimed to advance natural food packaging design by (i) developing and characterizing three NADES as green extraction media; (ii) obtaining bioactive extracts from Uxi bark and Jambolan leaves using these NADES; (iii) formulating babassu mesocarp-based coatings enriched with NADES citric acid extracts; and (iv) evaluating their application on cherry tomatoes.

## 2. Materials and Methods

### 2.1. Material

Yellow uxi bark and Jambolan leaves were purchased at a local market (Rio de Janeiro, RJ, Brazil). Babassu mesocarp flour (OKKA, Fortaleza, Brazil) was purchased online. For the preparation of NADES, choline chloride, citric acid, urea and glucose were supplied by Sigma-Aldrich (St. Louis, MO, USA). Standard solutions of Al, Cr, Ca, Cu, Fe, K, Mg, Mn, P, S, Sr, Zn, Se, Co, Cd, Pb, Cd, Fe, and Mn were obtained from Specsol^®^ (São Paulo, Brazil). An ICP-OES model iCAP 6300, equipped with a Mira Mist nebulizer (Burgener Research Inc., Mississauga, ON, Canada) and a cyclone spray chamber (Thermo Scientific, Waltham, MA, USA), was utilized for the multi-element determination. The following chemical reagents were used for the analyzes and quantifications of the antioxidants: 2,2-difenil-1-picrilhidrazila (DPPH), Folin–Ciocalteu phenol reagent, Nile red (Sigma-Aldrich, St. Louis, MO, USA), anhydrous gallic acid and absolute ethyl alcohol P.A. (Êxodo Científica, Sumaré, SP, Brazil).

### 2.2. Characterization of Uxi Barks and Jambolan Leaves

Uxi bark and Jambolan leaves were ground, and sieved through a 100-mesh sieve. These powders were stored refrigerated and protected from light for 24 h prior to extract preparation. The centesimal composition [29], multielement determination [30], and color parameters [31] of both powders were analyzed, detailed methodologies are provided in the Supplementary Materials.

### 2.3. NADES Preparation and Characterization

NADES were prepared by mixing the hydrogen bond acceptor (HBA), choline chloride, with three different hydrogen bond donors (HBDs), citric acid (CC-CA), urea (CC-U), and glucose (CC-G), by heating (60 °C) and stirring (2 h) until homogeneous and transparent liquids were obtained. For the formation of CC-CA and CC-G, both species were mixed in a 1:1 ratio, adding 30% (*w*/*w*) water. CC-U, in turn, was prepared by mixing the components in a 1:2 molar ratio with 30% (*w*/*w*) water [32]. NADES were stored at 25 °C and characterized in triplicate for their pH (determined at 25 °C with a portable pH meter (K39-220, Kasvi, Pinhais, SP, Brazil) and density by pycnometry at 25 °C, weighing the mass of NADES in 5 mL pycnometers. In addition, the viscosity of NADES was determined using a controlled-voltage rheometer (AR-1000N, TA Instruments, New Castle, DE, USA) and a Peltier system for temperature control. Steady-state flow measurements were performed using a cone plate geometry with a cone angle of 2°, a diameter of 40 mm, a gap of 55 μm, a fixed shear rate of 1 s^−1^ and a temperature between 20 and 50 °C [33]. Finally, the polarity of NADES was determined by solvatochromic method using a UV-Vis spectrophotometer (UV-M51, BEL^®^ Engineering, Monza, MB, Italy), according to the method described by Fernandes et al. [33] with slight modifications [16]. NADES (1:200 dye: solution) was added to an ethanolic solution of Nile Red dye 1 mg ml^−1^ and a wavelength scan was performed from 800 to 200 nm. The maximum absorption wavelength (λmax) of each solution was used to calculate the polarity parameter (E_NR_) in triplicate [34].

### 2.4. Production and Characterization of NADES Extracts from Uxi Bark and Jambolan Leaves

Extracts of Uxi bark (YU) and Jambolan leaves (JL) were prepared with NADES (CC-CA; CC-U; and CC-G). 1 g of YU or JL were mixed with 19 g of NADES in an ultrasonic bath (SSBU/3.8, Solidsteel, São Paulo, Brazil) at 45 °C for 2 h. After extraction, the mixtures were filtered. To compare the extraction efficiency of NADES, the same conditions were used with a conventional solvent EtOH/H_2_O 60% (*v*/*v*) [16]. Table 1 shows the identification of the extracts and the type of solvent used to extract the YU and JL.

The pH of the extracts was determined at 25 °C using pH strips (universal paper type, precision of 1.0 pH unit), and the color parameters (L*, a*, b*) were determined using a colorimeter (3nh, Colorimeter Spectrometer Y53020, Shenzhen, China). The chroma value (C_ab_*) was calculated and the color difference (∆E*) was calculated, considering ∆E* between the extract obtained with the conventional solvent EtOH/H_2_O 60% (L*_0_, a*_0_, b*_0_) and the other extracts obtained with NADES from the same samples (YU and JL) [35].

The total reducing capacity was determined using the Folin–Ciocalteu method [16]. The absorbance was measured using a UV/VIS spectrophotometer (Libra S22, Biochrom, Cambridge, UK). Gallic acid (5–100 µg mL^−1^) was used as a standard, and the results were expressed as mg of gallic acid equivalent (mg GAE) per mL of extract. Antioxidant activity was evaluated using the DPPH radical scavenging assay. The diluted extracts (1:20 in ethanol) were reacted with the DPPH solution according to the methodology adapted from Souza et al. [36] and Bertolo [17].

The antimicrobial activity was initially assessed by measuring the diameter of the inhibition zones (mm) against four bacterial strains: (*Staphylococcus aureus*-ATCC 25923, *Escherichia coli*-ATCC 25922, *Pseudomonas aeruginosa*-ATCC 27853 and *Salmonella* sp.-ATCC 14029). The inoculum of the strains was prepared in a saline solution until reaching similar turbidity for a concentration of 0.5 McFarland. The halo diameters were measured after incubating the plates on Müller–Hinton agar at 37 °C for 24 h [37]. A sterile paper disk impregnated with sterile distilled water served as the negative control, while azithromycin (15 μg), imipenem (10 μg), and ciprofloxacin hydrochloride (5 μg) were used as positive controls. Pure NADES were also evaluated under the same conditions.

Based on the disk diffusion assay, total reducing capacity, and antioxidant potential, the YU-CA and JL-CA extracts were selected for minimum inhibitory concentration (MIC) and minimum bactericidal concentration (MBC) analysis. Dilutions in Mueller–Hinton broth were prepared to determine the MIC and MBC [38,39]. Mueller–Hinton broth inoculated with bacteria at a final concentration of 5 × 10^5^ CFU mL^−1^ in a 96-well microplate was incubated at 37 °C for 24 h. Then, 20 μL of 2,3,5-Triphenyl Tetrazolium Chloride (3 g 100 mL ^−1^) was added to each well as a color indicator and incubated for 1 h. The MIC was defined as the lowest concentration of extract that inhibited visible growth (no color change to red). From wells without color change, 7 μL was inoculated onto the surface of Mueller–Hinton agar and incubated at 37 °C for 24 h. The MBC was defined as the lowest concentration at which no microbial growth was observed.

### 2.5. Biopolymer Coating: Development, Characterization and Application on Cherry Tomatoes

Coating suspensions containing babassu mesocarp (4 g 100 g^−1^ of suspension) were homogenized using a magnetic stirrer (AREX Heating Magnetic Stirrer, Velp Scientifica, Usmate Velate, Italy) for 30 min. Subsequently, the suspensions were heated to 90 °C for 15 min. After cooling the suspension to 60 °C, YU-CA or JL-CA extracts (100 g of extract 100 g^−1^ of babassu mesocarp) were added and stirred for an additional 10 min. Two coatings were developed: C-YU-CA (coating with NADES-Uxi bark extract) and C-JL-CA (coating with NADES-Jambolan leaf extract).

For analysis of total reducing capacity and antioxidant potential, the coatings were subjected to extraction described for Barone et al. [40]. The coatings were evaluated for (i) total reducing capacity and (ii) antioxidant activity by the DPPH method and the color parameters as described in Section 2.4. The dilution in Mueller–Hinton broth was performed to determine the minimum bactericidal concentration (MBC) following the method of Filho et al. [39]. The steps of the analysis were conducted as detailed in Section 2.4.

For the coating, cherry tomatoes of uniform size and ripeness were selected, with no visible mechanical damage or deterioration. The ripeness stage was defined according to the USDA tomato ripeness classification, established as stage 6 (red), which corresponds to typical commercial ripeness [41,42]. For the application of the coatings, the cherry tomatoes were divided into three groups: Control (distilled water), C-YU-CA (coating with NADES–Uxi bark extract), and C-JL-CA (coating with NADES–Jambolan leaf extract). The fruits were washed, sanitized in 0.1% sodium hypochlorite for 10 min, and air-dried. Coatings were applied by double immersion (20 s immersion, 20 min drying, repeated once) [43]. After coating, all samples were air-dried and stored in a BOD incubator (TE-371/240 L, Piracicaba, Brazil) at 25 ± 1 °C for 9 days. Tomato quality was assessed for the following: (i) weight loss (%); (ii) firmness (N), measured with a texture analyzer (TX-700, Lamy Rheology, Champagne-au-Mont-d’Or, France); (iii) color, expressed as the redness index (a*/b*) following the protocol in Section 2.4 [26]; (iv) internal quality, including titratable acidity [44] and pH, determined by homogenizing 10 g of tomato in 50 mL water, filtering, and measuring with a digital pH meter (R-TEC-7-MP, Tecnal, Piracicaba, Brazil) [45]; and (v) microbiological quality, evaluated during 9 days of storage for *Salmonella* sp., *Escherichia coli*, total plate count (TPC), and yeast and mold counts, according to Silva et al. [46]. All quality analyses were performed in triplicate on days 0, 3, 7, and 9. For sensory analysis, bruschettas were prepared with cherry tomatoes coated the day before each test. The tomatoes (100 g) were washed to remove the coating, quartered, and sautéed with 5 g olive oil, 2 g garlic, and 1 g salt for approximately 3 min. Portions (5 g) were served on naturally fermented bread and topped with 0.5 g dried basil. Sensory evaluation was performed with 110 untrained panelists (81.6% women, 18.4% men, aged 18–47 years). Overall acceptability was assessed using a 9-point hedonic scale ranging from “like extremely” to “dislike extremely” [47]. Panelists could also provide open comments. Participants completed a questionnaire on cherry tomato consumption and waste habits (questions in Table S2; responses in Figures S2 and S3, Supplementary Data). The study was approved by the Research Ethics Committee of the Pedro Ernesto University Hospital, Rio de Janeiro State University, Brazil on 6 June 2018 (approval number 2694935).

### 2.6. Statistical Analysis

Results were expressed as mean ± standard deviation. Data were analyzed with GraphPad Prism version 6.0 (Graph Pad Software, Inc., San Diego, CA, USA) using one-way analysis of variance (ANOVA) and Tukey’s test, with statistical significance set at the 5% level.

## 3. Results and Discussion

### 3.1. NADES Preparation and Characterization

All NADES formulations resulted in homogeneous and stable liquids at 25 °C with no recrystallization observed. Table 2 presents their physicochemical properties.

The pH values (Table 2) showed wide variation, ranging from highly acidic (CC-CA) to neutral (CC-U). This variation is primarily influenced by the structure and nature of the hydrogen bond donor (HBD) used in each formulation [48]. Citric acid, glucose, and urea were responsible for the acidic (pH 0 ± 0, CC-CA), slightly acidic (pH 5 ± 0, CC-G), and neutral (pH 7 ± 0, CC-U) pH values, respectively. In general, NADES with lower pH values (<4.0), such as CC-CA, tend to exhibit higher extraction efficiency for bioactive compounds. This is attributed to their enhanced ability to donate protons and accept electrons, facilitating the solubilization of both polar and non-polar compounds [15,48].

Density is an important physicochemical parameter and is influenced by several factors, including the nature and molar ratio of the hydrogen bond acceptor (HBA) and donor (HBD), as well as the amount of water added to the eutectic mixture [17]. All NADES investigated (Table 2) exhibited densities higher than that of water (~1.0 g cm^−3^). At room temperature (25 °C), the densities of the choline chloride-based NADES ranged from 1.152 ± 0.037 g cm^−3^ for CC-U, to 1.239 ± 0.009 g cm^−3^ for CC-G and 1.234 ± 0.038 g cm^−3^ for CC-CA. Only slight differences were observed among the NADES densities, which can be attributed to all the formulations containing the same water content (30%). This finding is in agreement with Bertolo et al. [17], who also reported minimal variation in the densities of NADES synthesized with a fixed water content of 20%.

Viscosity is one of the main technological limitations for the use of NADES as solvents for bioactive compounds extraction. The high viscosity of NADES can decrease solute diffusion, reduce solid–solvent interaction, and consequently reduce extraction performance [15]. To overcome this limitation, the addition of water and the increase in temperature are commonly employed strategies to reduce viscosity [15]. The optimal water content in NADES ranges between 20% and 40%. However, water concentrations above 50% may disrupt the hydrogen bonds, compromising the eutectic structure [49]. Therefore, a water content of 30% (*v*/*v*) was selected for all NADES formulations in this study to reduce viscosity while maintaining structural integrity, aiming to enhance the extraction efficiency of bioactive compounds.

Among the NADES evaluated, CC-G (18.375 ± 0.430 mPa·s) exhibited the highest viscosity at room temperature (25 °C), followed by CC-CA (12.566 ± 0.111 mPa·s) and CC-U (3.742 mPa·s) (*p* ≤ 0.05). All NADES in this study showed higher viscosities than conventional solvents such as ethanol (~1.082 mPa·s) and water (~0.890 mPa·s), as previously reported in the literature [15,48]. Benvenutti et al. [48] synthesized a choline chloride/citric acid NADES (1:1) without water addition with a viscosity of 14.480 Pa·s.

**Figure 1:**
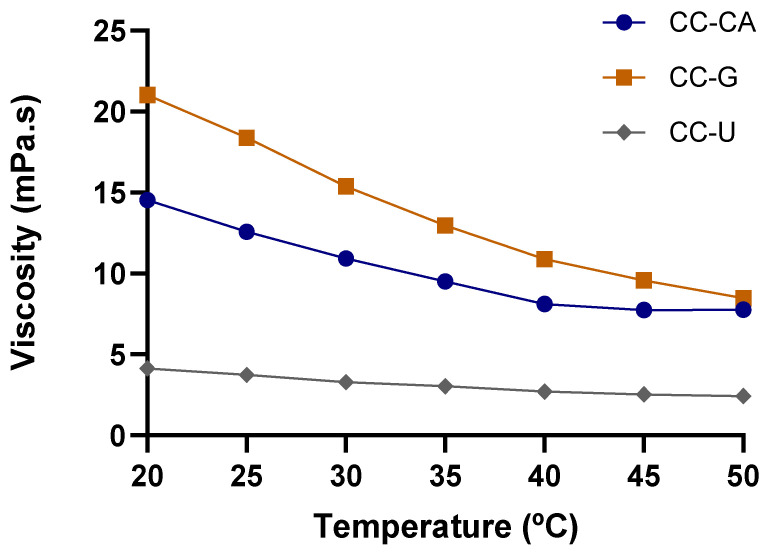
Curves of viscosity at 1 s^−1^ versus temperature ranging from 20 to 50 °C of NADES based on choline chloride–acid citric (CC:CA), choline chloride–glucose (CC-G) and choline chloride–urea (CC-U).

The viscosity decreased significantly as the water content increased [50]. In this sense, the main reason for the divergence between our results and those of other studies can be explained by the percentage of water added. A viscosity study was plotted for the prepared NADES (Figure 1), revealing an exponentially decaying viscosity–temperature profile, which has also been reported by other authors [15,33]. In all cases, heating (20 to 50 °C) reduced viscosity by more than 40%, reaching up to 60% reduction for CC-G, highlighting the potential of temperature modulation to improve NADES performance in extraction processes.

Polarity is a fundamental characteristic of NADES as solvents, as it influences both the quantity and the profile of bioactive compounds [16]. In this study, the polarity of the NADES was evaluated using Nile Red dye as a solvatochromic probe, which measures variations in the E_NR_ parameter of the dye diluted in the solvent. This parameter is inversely proportional to polarity: less polar solvents exhibit higher E_NR_ values [15].

Among the NADES analyzed, CC-CA showed the lowest E_NR_ value (44.6 ± 0.1 kcal mol^−1^), being the most polar (*p* < 0.05), whereas CC-G presented the highest value (49.9 ± 0.1 kcal mol^−1^), indicating it was the least polar. Organic acid-based NADES generally exhibit higher polarity, with E_NR_ values ranging from 44 to 48 kcal mol^−1^, similar to water (E_NR_ = 48.2 kcal mol^−1^). This higher polarity can be attributed to the fact that acids (HBD) have a better capacity for hydrogen bonding to choline chloride (HBA) compared to other HBDs. In contrast, NADES formulated with sugars as HBDs tend to exhibit lower polarity, with E_NR_ values ranging from 49.7 to 50.7 kcal mol^−1^, close to that of methanol (E_NR_ = 51.9 kcal mol^−1^) [50,51].

### 3.2. Characterization of NADES–Uxi Bark Extract and NADES–Jambolan Leaves Extract

Once prepared and characterized, the NADES were employed for the extraction of YU and JL. Figure S1 presents the visual appearance of the plant powders. Colorimetric analysis revealed significant visual differences between the YU and JL powders (Table S1 and Figure S1, Supplementary Materials). A ΔE value of 12.3 indicates a highly noticeable color difference perceptible to the naked eye [52], confirming that the two powders have distinctly different visual characteristics. Both plant powders exhibited a high carbohydrate content (YU: 81.58 g 100^−1^; and JL: 77.85 g 100^−1^) (Table S1, Supplementary Materials). Multi-element analysis showed the presence of cadmium (Cd), chromium (Cr), cobalt (Co), and lead (Pb) at concentrations below the detection limits of the analytical method: 3 µg g^−1^ for Cd, 2 µg g^−1^ for Cr, 3 µg g^−1^ for Co, and 21 µg g^−1^ for Pb (Table S1, Supplementary Materials). Heavy metal pollution in plants has seriously compromised quality and safety due to its toxicity and carcinogenic potential, potentially causing serious health problems. To address this issue, controlling heavy metal levels in plants is essential [53].

The comparison of NADES-based extracts from YU with the ethanolic extract revealed ΔE values ranging from 0.59 to 3.07. The differences between YU-CA and YU-U in relation to the ethanolic extract (YU-E) were classified as “slight differences,” whereas the comparison between YU-G and YU-E indicated a “noticeable difference” [52]. The chromatic saturation (C* = 0.22–0.65) was low, indicating YU extracts had a low color intensity. For JL extracts, when comparing NADES with the ethanolic extract, the only ΔE above 3 was observed between JL-G and JL-E (6.25), which corresponds to an “appreciable difference” [52]. Chromatic saturation was also low (C* = 0.13–2.01), suggesting a soft and low-intensity coloration. In general, the changes observed in the color parameters may be related not only to the pigment content but also to the stability of these pigments in relation to pH, temperature, and solvent composition. The different polarities and pH values of NADES influence the extraction of pigments, such as anthocyanins and carotenoids, directly affecting lightness (L*), hue (a* and b*), and chroma (C*), and impact the overall visual perception of the extracts [54,55].

The pH of the extracts showed a similar pattern to that observed in their respective NADES, varying according to the nature of the hydrogen bond donor (HBD) used (Table 3). Extracts prepared with CC-CA (YU-CA and JL-CA) exhibited the lowest pH values (around 0–1), reflecting the acidity imparted by citric acid. In contrast, extracts obtained with CC-U displayed higher pH values (6–7), consistent with the lower acidity of this component. Ethanolic extracts presented intermediate pH values (4–5). A similar behavior was observed for the NADES based on choline chloride and lactic acid (pH 1.4) and for the annatto extract obtained with this solvent (pH 1.28), indicating that the pH value is mainly determined by the extraction solvent’s pH, while also being influenced by the plant species used and the chemical composition of the extract [15].

#### Bioactivity Screening Assays

**Figure 2:**
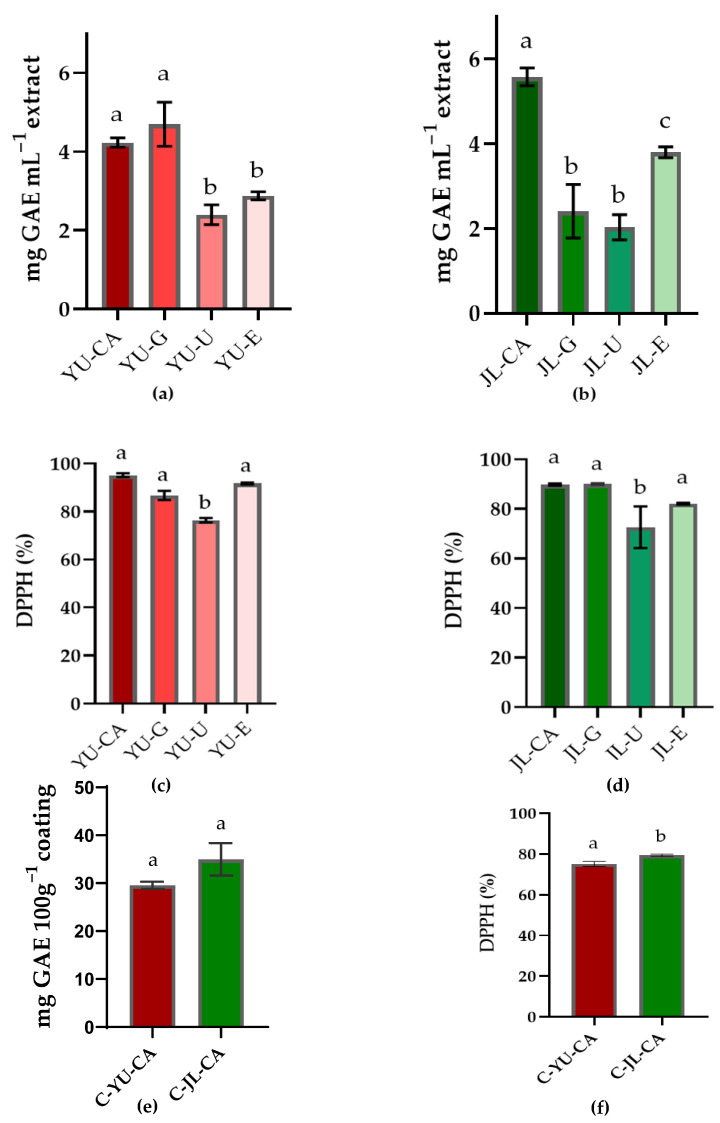
Total reducing capacity (in mg GAE ml^−1^ extract) (**a**,**b**,**e**) and DPPH (%) (**c**,**d**,**f**) for Extracts of Uxi bark using NADES (choline chloride-citric acid (YU-CA), choline chloride-glucose (YU-G) and choline chloride-urea (YU-U)), and EtOH/H_2_O 60% (YU-E); for Jambolan leaves using NADES choline chloride-citric acid (JL-CA), choline chloride-glucose (JL-G) and choline chloride-urea (JL-U), and EtOH/H_2_O 60% (JL-E), and for coating with NADES-Uxi extract (C-YU-CA) or NADES-Jambolan leaf extract (C-JL-CA). Bars with the same superscript letter are statistically equal (*p* > 0.05) according to ANOVA and Tukey’s test (*p* < 0.05).

The analysis of the total reducing capacity results indicated that the extraction efficiency of total phenolic compounds from YU and JL was largely dependent on the type of NADES used (Figure 2a,b). For YU, extracts obtained with CC-CA (YU-CA) (4.23 ± 0.1 mg GAE mL^−1^) and CC-G (YU-G) (4.69 ± 0.15 mg GAE mL^−1^) showed the highest total reducing capacity. In contrast, the lowest total reducing capacity were found in the extract obtained with CC-U (YU-U), which reached 2.4 ± 0.05 mg GAE mL^−1^. Regarding JL, the extract obtained with CC-CA (JL-CA) also stood out, exhibiting the highest total reducing capacity (5.57 ± 0.2 mg GAE mL^−1^). Conversely, the extracts obtained with CC-G (JL-G) and CC-U (JL-U) presented the lowest total reducing capacity values, 2.41 ± 0.1 and 2.03 ± 0.05 mg GAE mL^−1^, respectively. The variations in extraction efficiency among different NADES formulations for phenolic compounds can be explained by factors such as hydrogen bonding interactions, polarity matching, and viscosity effects, as supported by previous studies on NADES–solute interactions [15,17]. In this study, NADES formulations containing citric acid as the HBD generally produced extracts with higher total reducing capacity compared to those containing urea or glucose. This result can be attributed to the carboxyl groups in citric acid, which are strong hydrogen bond donors capable of interacting with the hydroxyl groups of phenolic compounds [32,56].

The results of the antioxidant activity (DPPH-%) are shown in Figure 2c,d. NADES-based extracts formulated with urea (YU-U-76.41 ± 0.92%; JL-U-72.65 ± 8.43%) exhibited lower performance compared to the other extracts. In contrast, extracts prepared with citric acid (YU-CA-95.15 ± 0.8%; JL-CA-89.82 ± 0.46%), glucose (YU-G-86.72 ± 0.35%; JL-G-90.04 ± 0.35%), or ethanol (YU-E-91.7 ± 0.3%; JL-E-82.13 ± 0.3%) stood out with higher antioxidant activities. It is worth noting that these DPPH inhibition values (%) were obtained with extracts diluted 20 times. The results reveal a trend similar to that observed for total reducing capacity assays, which could be linked to the interactions between NADES molecules and bioactive compounds with antioxidant properties. Previous studies have also reported a strong relationship between phenolic content and antioxidant activity [17,19].

Given the potential application of the extracts as natural food additives, their antimicrobial activity was evaluated, and the results are presented in Table 4.

The antimicrobial activity of YU and JL extracts varied according to the extraction solvent used and the bacterial strain tested. For YU, the extract obtained with CC-CA (YU-CA) exhibited extremely sensitive responses against *S. aureus* (30 ± 2.6 mm) and *P. aeruginosa* (34.33 ± 1.15 mm) and very sensitive responses against *Salmonella* spp. (15.17 mm). This performance was significantly superior to that obtained with the other tested solvents. However, when compared with the isolated CC-CA NADES, the YU-CA extract showed greater activity only against *P. aeruginosa*. Extracts obtained using glucose, urea, or ethanol showed no inhibition zones against *E. coli*, *P. aeruginosa* and *Salmonella* sp., suggesting that these solvents selectively extracted compounds with predominant activity against Gram-positive bacteria. Similarly, for JL, the JL-CA extract exhibited superior performance, with extreme sensitivity against *S. aureus* (26 ± 3.61 mm), *P. aeruginosa* (31 ± 2.59 mm), and *Salmonella* spp. (20.5 ± 2.06 mm). In this study, the citric acid-based NADES played a significant role in the antimicrobial activity of YU-CA and JL-CA, likely due to its acidic pH. Citric acid is known to inactivate bacteria by destabilizing the outer membrane or chelating essential metals from the growth medium [57].

The use of NADES has been increasingly recognized for its superior performance compared to conventional solvents. Wu et al. [56] demonstrated their effectiveness in extracting bioactive compounds from cotton by-products, with CC-CA NADES being particularly potent, exhibiting a fungal inhibition rate of 78.42%. Similarly, Bertolo et al. [17] reported the successful extraction of essential oils from orange peels with high antimicrobial activity. The results of the present study clearly demonstrate that potent plant-derived antimicrobial compounds can be obtained through green extraction methods such as NADES, offering advantages over conventional solvent extraction. This represents a promising potential for the development of novel natural antimicrobials, with applications ranging from biomedical to food sectors. Based on these findings, citric acid-based NADES extracts (YU-CA and JL-CA) were selected for subsequent stages of the study.

### 3.3. Biopolymer Coating: Development, Characterization and Application on Cherry Tomatoes

Table 4 presents the results of the MIC/MBC analysis for the extracts and the MBC for their respective coatings. It is important to note that it was not possible to determine the MIC in the coatings due to a methodological limitation. The color change to red caused by the indicator 2,3,5-triphenyl tetrazolium chloride in the presence of microbial growth could not be observed. The extracts obtained with CC-CA NADES (YU-CA and JL-CA) exhibited species-dependent profiles. The most sensitive bacterium was *P. aeruginosa*, inhibited by extracts at 1.05 mg mL^−1^ (YU-CA) and 0.52 mg mL^−1^ (JL-CA). Incorporation of the extracts into the coatings (C-YU-CA and C-JL-CA) led to a reduction in antimicrobial potency, as indicated by increased MBC values for all tested bacteria. Nevertheless, the coatings retained activity, particularly against *P. aeruginosa* (93.75 mg mL^−1^ for C-YU-CA and 187.5 mg mL^−1^ for C-JL-CA), although at considerably higher concentrations than the free extracts. The decrease in antimicrobial activity may be associated with interactions between the biopolymer matrix and phenolic compounds, such as hydrogen bonding and physical entrapment [58]. These interactions can limit the mobility, diffusion, and immediate availability of active compounds at the food surface. Consequently, the release kinetics of phenolic compounds from the coating may be slowed, resulting in lower apparent antimicrobial activity compared to the free extracts [58]. Furthermore, the antimicrobial activity of the coatings can be attributed to the bioactive compounds present in the extracts, and is positively correlated with the extract concentration in the coatings [58].

As shown in Figure 2e, no statistical difference was observed between the coatings regarding total reducing compound content. In contrast, the free radical scavenging capacity determined by the DPPH method was statistically higher (*p* > 0.05) for the C-JL-CA coating (79.4% ± 0.55) compared to C-YU-CA (75.14% ± 1.22) (Figure 2f). Antioxidant properties in food coatings play a key role in preservation, as they help mitigate oxidative damage and delay the aging process [59]. The coatings showed similar colorimetric parameters (ΔE = 1.04), with chroma values of 9.66 for C-YU-CA and 9.40 for C-JL-CA.

**Figure 3:**
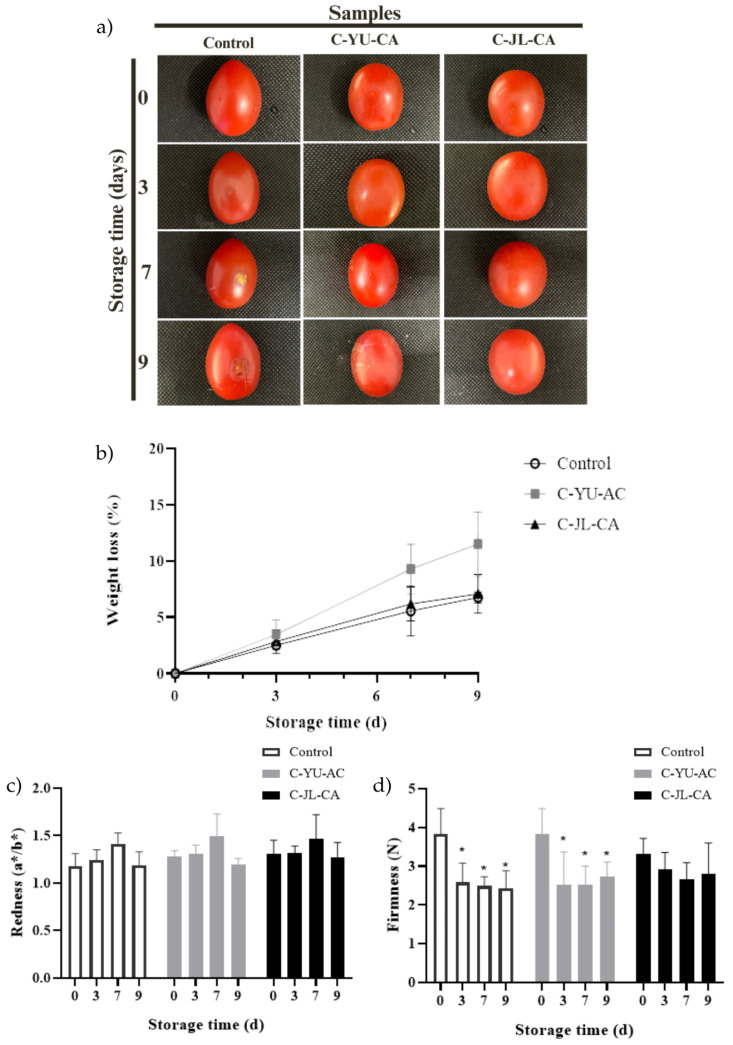
Visual images of cherry tomato samples (C-YU-CA, C-JL-CA, and control) during 9 days of storage (**a**). Changes in (**b**) weight loss (%), (**c**) redness (a*/b*), and (**d**) firmness (N) of cherry tomatoes C-YU-CA, C-JL-CA and control during storage. For each treatment (C-YU-CA, C-JL-CA, and control), bars followed by asterisks (*) differ significantly from day 0 by the ANOVA and Tukey test (*p* < 0.05). Abbreviations: C-YU-CA, coating cherry tomatoes with NADES-Uxi extract; C-JL-CA, coating cherry tomatoes with NADES-Jambolan leaf extract.

Appearance is directly related to the external quality of the fruit and has a major influence on sales and consumer acceptance [60]. In order to evaluate the effect of C-YU-CA and C-JL-CA on the preservation of cherry tomato appearance, a storage experiment was conducted at room temperature (25 °C ± 1 °C) for 9 days. As shown in Figure 3a, in the control group, after the 7th day, the fruit skin showed clear signs of deterioration, with the process intensifying in the following days. In the samples coated with C-YU-CA, also from the 7th day onwards, the tomatoes began to show signs of wilting. On the other hand, the C-JL-CA coating significantly delayed the softening and deterioration of the tomatoes throughout the storage period. The coating containing JL extract with NADES-CA was effective in preserving the visual quality of cherry tomatoes, also demonstrating a certain antibacterial effect by acting as a barrier against infection by external microorganisms [60].

Weight loss is one of the main indicators of quality during cherry tomato storage. This parameter is directly associated with natural water loss and nutrient consumption. As shown in Figure 3b, all samples exhibited a gradual reduction in weight during the storage period, which is expected and consistent with the literature [26,60]. However, the coated samples showed a lower rate of weight loss from the 7th day onwards, with no significant difference between the values recorded on the 7th and 9th days (*p* < 0.05). These results indicate that the coatings applied significantly slowed the weight loss of cherry tomatoes during the storage period, acting as an effective physical barrier against water loss and oxygen entry. Thus, they contributed to slowing the consumption of nutrients associated with cellular respiration, as well as reactions related to fruit senescence [60,61].

Changes in firmness are important indicators of the stages of ripeness and sensory acceptance of the fruit [26]. When evaluating fruit firmness during storage (Figure 3d) a similar trend was observed to that seen for weight loss in the control samples and those coated with YU-CA. The control group showed a progressive decrease in firmness over the 9 days of storage. In turn, tomatoes coated with YU-CA showed a reduction in firmness until the 7th day, with no significant difference between the values obtained on days 7 and 9 (*p* > 0.05). On the other hand, tomatoes coated with JL-CA showed no significant changes in firmness throughout the storage period. This result suggests that the JL extract with NADES-citric acid present in the C-JL-CA may have acted to inhibit the degradation of pectin and hemicellulose by specific enzymes, delaying softening and contributing to the preservation of the cellular structure of the fruits throughout storage [61].

Color is a crucial quality attribute of tomatoes and directly influences their acceptability to consumers. Color changes during tomato ripening occur mainly due to chlorophyll degradation and lycopene synthesis, a compound responsible for the characteristic red color of the fruit [45]. As shown in Figure 3c, the redness (a*/b*) of tomatoes was not significantly altered in any of the treatments during the 9 days of storage. Consequently, variations in lycopene concentration were also considered insignificant during this period [26]. In addition, the redness of the cherry tomatoes in the control group and the coated samples showed no statistical differences, which is considered a positive result, since the application of coatings with film-forming solutions can, in some cases, promote changes in color and increase the opacity of the fruits, impairing their visual appearance [45,61].

The pH of the control sample showed a significant increase (*p* > 0.05) from day 0 to day 3, rising from 3.89 ± 0.03 to 4.42 ± 0.19. In contrast, the pH levels of the coated fruits remained stable over time (Table 5). No statistically significant differences among treatments (*p* > 0.05) were observed. Similarly, titratable acidity (TA) values did not differ significantly between treatments (*p* > 0.05). However, a decrease in acidity was observed in the control group starting from day 3 of storage, and this trend continued through days 7 and 9. In tomatoes coated with C-YU-CA, no significant changes (*p* > 0.05) in TA values were detected. Tomatoes treated with C-JL-CA showed a decrease in acidity only on day 9. Changes in acidity, along with an increase in pH, are naturally expected during tomato ripening. However, the smaller variation in TA values during storage in coated fruits suggests that the ripening process was delayed [26]. Previous studies have shown that applying coatings to fruit surfaces can reduce the transpiration rate, thereby limiting the consumption of organic acids such as citric acid [43].

Microbiological analyses were carried out to assess the hygienic-sanitary quality of the tomatoes over the 9-day storage period (Table 5). The results showed the absence of *Salmonella* spp. in 25 g and no growth of typical *E. coli* colonies in any of the samples. For total plate counts and yeast and mold analyses, microbial growth was observed only on day 9. The control sample showed significantly higher total plate counts (*p* > 0.005) compared to the C-YU-CA and C-JL-CA samples. In the yeast and mold analysis, no colony growth was observed in the C-YU-CA samples throughout the storage period. However, both the control and C-JL-CA samples showed a value of 2 × 10^1^ CFU g^−1^ on day 9. Other studies have also reported a positive effect of edible coatings on the microbiological quality of tomatoes [26,62]. Kumar et al. [43] attributed the lower total plate counts in coated tomatoes to the antimicrobial activity of clove oil. In the present study, the positive effect of the coatings on the microbiological quality of the cherry tomatoes can be attributed to the NADES (choline chloride: acid citric) extracts of YU and JL incorporated into the coatings, due to their antimicrobial properties.

**Figure 4:**
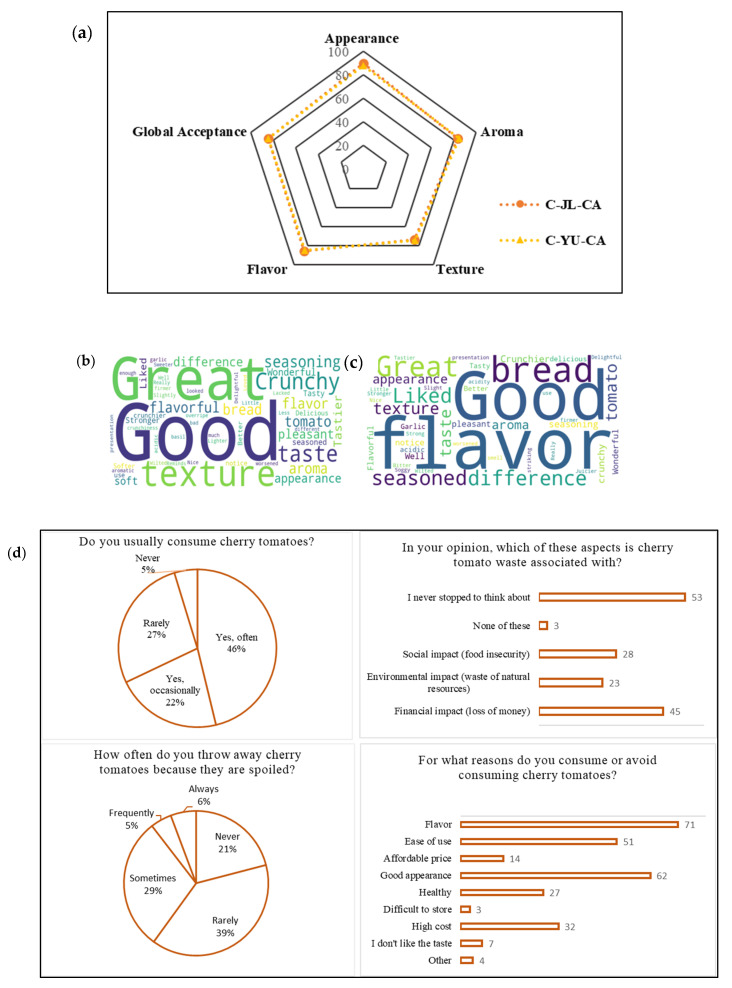
(**a**) Average score of the acceptance of the bruschetta with tomatoes coated with NADES-citric acid extract of Uxi bark (C-YU-CA) and with NADES-citric acid extract of Jambolan leaves (C-JL-CA) and word cloud of tasters’ comments. (**b**) Bruschetta with tomatoes coated with NADES-citric acid extract of Jambolan leaves and (**c**) bruschetta with tomatoes coated with NADES-citric acid extract of Uxi bark; (**d**) panelists ‘ responses to the questionnaire on cherry tomato consumption and waste habits.

The sensory analysis of bruschetta prepared with tomatoes coated with CC-CA NADES extract of YU (C-YU-CA) and with CC-CA NADES–JL extract (C-JL-CA) (Figure S2) was carried out to evaluate whether the coating applied to cherry tomatoes influences the acceptability of untrained panelists. Removing the coating by washing before sensory evaluation limits the study, as it prevents a direct assessment of the coating’s sensory impact, including any foreign or residual flavors. Instead, the focus shifts to how the coating affects tomato quality after storage. The desired organoleptic properties accepted by consumers are directly related to the texture, flavor, and aroma of the products [63]. Therefore, sensory analysis and the assessment of consumer acceptability have become important tools in guiding the improvement of this study. Figure 4a presents the average score of the acceptance bruschetta for each evaluated sensory attribute. The results showed good acceptance by most panelists. The bruschetta made with C-YU-CA coated tomatoes achieved an average acceptability score of 83.2 ± 5.2%, while the bruschetta prepared with C-JL-CA-coated tomatoes obtained an average acceptability score of 83.4 ± 6%.

Texture was the attribute with the lowest acceptance for both samples, with 73% for C-JL-CA and 74% for C-YU-CA. Figure 4b,c) show the main comments made by the panelists about the bruschetta with C-YU-CA and C-JL-CA cherry tomatoes, respectively. Terms such as “crunchy bread” and “soft bread” were frequently mentioned, suggesting that the texture of the bread was a determining factor in the lower acceptance of this attribute. On the other hand, comments like “good taste” and “good flavor” were recurrent. Coatings can help preserve the natural aroma and flavor of fruits by keeping the volatile chemicals without evaporation [64].

The results of responses from panelists who participated in the sensory analysis of cherry tomatoes regarding their consumption habits and perceptions regarding waste of this food are available in the Supplementary Data (Figure 4d and Figure S3). Figure 4d shows the results of responses from panelists who participated in the sensory analysis of cherry tomatoes regarding their consumption habits and perceptions regarding waste of this food. Most participants consume cherry tomatoes frequently, 46% report consuming them frequently, and 22% report consuming them occasionally, totaling 68% of respondents. Among the reasons for consuming cherry tomatoes, taste is the most cited factor (71 responses), followed by good appearance (62 responses) and ease of use (51 responses). Based on self-reported perceptions in the sensory evaluation (not on an economic study), the predominant discouraging factor was high cost (32 responses). Frequency of consumption, education, and household income are just a few examples of the many variables that affect consumer preferences and choices. The familiarity of the panelists with the evaluated food, as well as the reasons that motivate them to consume it, reinforce that possible perceptible changes in the coated tomatoes did not compromise the acceptability of the bruschetta, suggesting that the product was within the expected sensory standard [65]. Based on the answers (Figure S3), it is clear that different strategies are adopted when cherry tomatoes start to spoil. The most prominent words, such as “I don’t let it spoil,” “Discard,” and “Trash,” indicate that many consumers prefer to avoid waste, yet still choose to discard the food at more evident signs of deterioration. The results of this questionnaire reveal that although cherry tomatoes are well accepted sensorially and frequently consumed, waste still occurs and is often not critically reflected upon by consumers. According to Marwood et al. [66], consumer-generated food waste is rooted in multiple interconnected actions, behaviors, and habits. Understanding these factors and being able to discuss strategies that reduce losses and raise awareness toward more sustainable choices is considered an important tactic in efforts to reduce global food waste [66].

## 4. Conclusions

The present study demonstrated that NADES, particularly those formulated with citric acid, represent a promising greener alternative to harsh industrial solvents for the extraction of bioactive compounds from Uxi bark and Jambolan leaves. The physicochemical characterization revealed that the composition and polarity of NADES directly influence extraction efficiency, with citric acid-based solvents standing out by yielding higher phenolic contents, stronger antioxidant activity, and significant antimicrobial effects. The coating formulations containing the extracts retained both antioxidant and antimicrobial activity, although at a lower level than the extracts themselves. The edible coatings applied to cherry tomatoes helped delay mass loss, preserve firmness, and maintain microbiological quality during storage, without significantly affecting color parameters or compromising sensory attributes relevant to consumer acceptance. It should be noted that the preservation effects were parameter-dependent, and the coatings did not uniformly improve all quality attributes. These findings reinforce the applicability of NADES as a promising green solvent in the food industry, not only for the extraction of bioactive compounds but also for the development of functional coatings with potential benefits in the preservation of vegetables such as cherry tomatoes. Future studies should focus on optimizing extract concentration in coatings and evaluating their stability during extended storage periods, aiming to expand the industrial application of this technology.

## Figures and Tables

**Table 1:** Extract identification and solvent type for the Uxi bark and Jambolan leaf extraction.

Classical Solvent				
Extract ID	Sample		Solvent	
YU-E	Uxi bark		EtOH/H_2_O 60% (*v*/*v*).	
JL-E	Jambolan Leaves		EtOH/H_2_O 60% (*v*/*v*).	
**Natural Deep Eutectic Solvents (NADES)**				
**Extract ID**	**Sample**	**HBA**	**HBD**	**Molar ratio**
YU-CA	Uxi bark	Choline Chloride	Citric acid	1:1
YU-G		Choline Chloride	Glucose	1:1
YU-U		Choline Chloride	Urea	1:2
JL-CA	Jambolan leaves	Choline Chloride	Citric acid	1:1
JL-G		Choline Chloride	Glucose	1:1
JL-U		Choline Chloride	Urea	1:2

**Table 2:** Physicochemical properties of NADES based on choline chloride-citric acid (CC-CA), choline chloride-glucose (CC-G) and choline chloride-urea (CC-U).

Properties	CC-CA	CC-G	CC-U
pH at 25 °C	0 ± 0 ^c^	5 ± 0 ^b^	7 ± 0 ^a^
Density at 25 °C (g cm^−3^)	1.234 ± 0.038 ^a^	1.239 ± 0.009 ^a^	1.152 ± 0.037 ^c^
Viscosity at 25 °C (mPa s)	12.566 ± 0.111 ^b^	18.375 ± 0.430 ^a^	3.742 ± 0.115 ^c^
E_NR_ (kcal mol^−1^)	44.6 ± 0.1 ^c^	49.9 ± 0.1 ^a^	49.4 ± 0.1 ^b^

E_NR_: transition energy, λ_max_: maximum absorption wavelength. Means ± standard deviation (n = 3). ^a–c^ Different letters on the same line indicate significant differences according to Tukey’s test (*p* < 0.05).

**Table 3:** Colorimetric parameters (L, a* and b*), chroma value (C_ab_*), color difference (∆E*) pH and visual appearance of the extracts of Uxi bark and Jambolan leaves using NADES or EtOH/H_2_O 60%.

Samples	Extracts	L*	a*	b*	C_ab_*	ΔE	pH Range	Visual Appearance
Uxi bark	YU-CA	24.29 ± 0.04 ^a^	0.46 ± 0.05 ^a^	−0.13 ± 0.0 ^a^	0.45 ± 0.02 ^d^	0.61 ± 0.01 ^a^	0–1	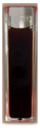
	YU-G	27.68 ± 1.52 ^b^	0.17 ± 0.09 ^b^	−0.13 ± 0.13 ^a^	0.41 ± 0.03 ^c^	4.04 ± 0.59 ^b^	3	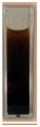
	YU-U	24.31 ± 0.03 ^a^	−0.24 ± 0.05 ^c^	−0.04 ± 0.04 ^a^	0.24 ± 0.04 ^b^	1.32 ± 0.07 ^a^	7–8	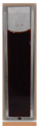
	YU-E	24.84 ± 0.04 ^a^	0.65 ± 0.02 ^a^	−0.05 ± 0.03 ^a^	0.65 ± 0.02 ^a^	-	4–5	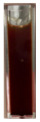
Jambolan leaves	JL-CA	25.07 ± 0.07 ^a^	0.66 ± 0.1 ^a^	0.87 ± 0.04 ^a^	1.1 ± 0.06 ^a^	2.02 ± 0.13 ^a^	0–1	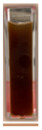
	JL-G	28.42 ± 0.58 ^b^	−0.03 ± 0.11 ^b^	−0.04 ± 0.18 ^b^	0.13 ± 0.04 ^b^	6.11 ± 0.11 ^b^	4–5	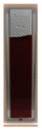
	JL-U	28.28 ± 0.58 ^b^	0.08 ± 0.05 ^b^	2.05 ± 0.55 ^c^	2.05 ± 0.55 ^a^	2.16 ± 0.66 ^a^	6–7	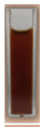
	JL-E	26.28 ± 0.05 ^a^	0.66 ± 0.11 ^a^	1.77 ± 0.05 ^c^	1.89 ± 0.04 ^a^	-	4–5	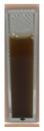

Values followed by different lowercase letters in the same column and within the same sample group (Uxi bark or Jambolan leaves) indicate a significant difference between the extracts, according to ANOVA and Tukey’s test (*p* < 0.05). Abbreviations: Extracts of Uxi bark: choline chloride-citric acid-YU-CA, choline chloride-glucose-YU-G, choline chloride-urea-YU-U, and EtOH/H_2_O 60%-YU-E. Jambolan leaves: choline chloride-citric acid-JL-CA, choline chloride-glucose-JL-G, choline chloride-urea-JL-U, and EtOH/H_2_O-60%.

**Table 4:** Antimicrobial activity by agar diffusion method, minimum inhibitory concentration (MIC) and minimum bactericidal concentration (MBC) of extracts of Uxi bark and Jambolan leaves using NADES or EtOH/H_2_O 60% and NADES based on choline chloride (control). Antimicrobial activity by MBC of the coating with NADES-Uxi extract and NADES-Jambolan leaf extract.

			*S. aureus*	*E. coli*	*P. aeruginosa*	*Salmonella*
Disk diffusion assay						
Extracts	Uxi bark	YU-CA	+++	+	+++	++
		YU-G	+	−	−	−
		YU-U	+	−	−	−
		YU-E	+	−	−	−
	Jambolan leaves	JL-CA	+++	+	+++	+++
		JL-G	++	−	−	−
		JL-U	+++	−	−	−
		JL-E	+	−	−	−
Solvent	NADES	CC-CA	+++	+	+++	++
		CC-G	−	−	−	−
		CC-U	−	−	−	−
Positive controls	Antibiotics	Azithromycin	+++	+++	+++	+++
		Imipenem	+++	+++	+++	+++
		Ciprofloxacin	+++	+++	+++	+++
Minimum inhibitory concentration						
Extracts	YU-CA (mg mL^−1^)		8.36	4.18	1.045	8.36
	JL-CA (mg mL^−1^)		8.36	8.36	0.52	8.36
Minimum bactericidal concentration						
Extracts	YU-CA (mg mL^−1^)		16.72	33.44	2.09	33.44
	JL-CA (mg mL^−1^)		33.44	66.87	2.09	16.72
Coatings	C-YU-CA (mg mL^−1^)		750	750	93.75	750
	C-JL-CA (mg mL^−1^)		375	750	187.5	750

The sensitivity of the extracts and NADES against the tested bacteria was classified according to the diameters of the inhibition zones (mm) as follows: “–” not sensitive for diameters < 8 mm; “+” sensitive for diameters between 9 and 14 mm; “++” very sensitive for diameters between 15 and 19 mm; and “+++” extremely sensitive for diameters > 20 mm [52]. Abbreviations: Extracts of Uxi bark: choline chloride-citric acid-YU-CA, choline chloride-glucose-YU-G a choline chloride-urea YU-U, and EtOH/H_2_O 60%-YU-E. Jambolan leaves: choline chloride-citric acid-JL-CA, choline chloride-glucose-JL-G, choline chloride-urea-JL-U, and EtOH/H_2_O-60%. NADES: chloride/citric acid-CC-CA, choline chloride/glucose-CC-G, and choline chloride/urea-CC-U; C-YU-CA, coating with NADES-Uxi extract; C-JL-CA, coating with NADES-Jambolan leaf extract.

**Table 5:** Effect of pH, titrable acidity (TA), *Salmonella* spp., *Escherichia coli*, Total Plate Count (TPC) and yeast and mold on C-YU-CA, C-JL-CA, and control cherry tomatoes during storage.

		Storage Time (d)			
Parameter	Sample	0	3	7	9
pH	Control	3.89 ± 0.03 ^aA^	4.42 ± 0.19 ^aB^	4.21 ± 0.19 ^aB^	4. 04 ± 0.11 ^aB^
	C-YU-CA	4.13 ± 0.18 ^aA^	4.21 ± 0.14 ^aA^	4.33 ± 0.18 ^aA^	4.16 ± 0.1 ^aA^
	C-JL-CA	4.01 ± 0.1 ^aA^	3.96 ± 0.02 ^aA^	4.31 ± 0.21 ^aA^	4.05 ± 0.16 ^aA^
TA (mg of citric acid 100 g^−1^ cherry tomatoes)	Control	3.99 ± 0.07 ^aA^	3.69 ± 0.48 ^aA^	2.79 ± 0.02 ^aB^	2.96 ± 0.11 ^aB^
	C-YU-CA	3.99 ± 0.59 ^aA^	4.14 ± 0.49 ^aA^	3.07 ± 0.53 ^aA^	2.77 ± 0.37 ^aA^
	C-JL-CA	3.82 ± 0.84 ^aA^	4.55 ± 0.45 ^aA^	2.68 ± 0.27 ^aA^	2.53 ± 0.55 ^aB^
*Salmonella* sp. in 25 g	Control	Absence	Absence	Absence	Absence
	C-YU-CA	Absence	Absence	Absence	Absence
	C-JL-CA	Absence	Absence	Absence	Absence
*E. coli* (CFU g^−1^)	Control	<10	<10	<10	<10
	C-YU-CA	<10	<10	<10	<10
	C-JL-CA	<10	<10	<10	<10
TPC (CFU g^−1^)	Control	<10	<10	<10	2 × 10 ± 0.0 ^a^
	C-YU-CA	<10	<10	<10	10 ± 0.0 ^b^
	C-JL-CA	<10	<10	<10	10 ± 0.0 ^b^
Yeast and mold (CFU g^−1^)	Control	<10	<10	<10	2 × 10 ± 0.0 ^a^
	C-YU-CA	<10^2^	<10^2^	<10^2^	<10^2^
	C-JL-CA	<10^2^	<10^2^	<10^2^	2 × 10 ± 0.0 ^a^

The data is presented as mean ± standard deviation (n = 3). For every parameter the data with different lowercase letters as superscript between rows (coating samples) are significantly different (*p* < 0.05) and those with different uppercase letters as superscript between columns (storage days) are significantly different. Abbreviations: C-YU-CA, coating cherry tomatoes with NADES-Uxi extract; C-JL-CA, coating cherry tomatoes with NADES-Jambolan leaf extract; TA, Titrable acidity; CFU, Colony Forming Unit.

## Data Availability

The original contributions presented in this study are included in the article/Supplementary Materials. Further inquiries can be directed to the corresponding author.

## Supplementary Materials

The following supporting information can be downloaded at https://www.mdpi.com/article/10.3390/foods15010074/s1, methodologies: proximate composition, multi-element determination, and colorimetric parameters of Uxi bark and Jambolan leaf powders. Table S1: Characterization of Uxi bark powder and Jambolan leaf powder. Figure S1: (A) Uxi bark powder < 100 mesh; (B) Color parameters of the uxi bark; (C) Jambolan leaf powder < 100 mesh; and (D) Jambolan leaf powders. Table S2: Questionnaire questions about cherry tomato consumption habits and perceptions of waste. Figure S2: Coated tomatoes with (a) NADES-Uxi extract (C-YU-CA) and (c) NADES-Jambolan leaf extract (C-JL-CA), and their respective bruschettas (b,d). Figure S3: Word cloud of tasters’ comments (Answer to the question: “What do you usually do when you notice that your cherry tomatoes are starting to spoil?”) [29,30,31].

## References

[1] Dash D.R., Singh S.K., Singha P. (2024). Bio-Based Composite Active Film/Coating from Deccan Hemp Seed Protein, Taro Starch and Leaf Extract: Characterizations and Application in Grapes. Sustain. Chem. Pharm..

[2] Anushikha, Deshmukh R.K., Kunam P.K., Gaikwad K.K. (2023). Guar Gum Based Flexible Packaging Material with an Active Surface Reinforced by Litchi Shell Derived Micro Fibrillated Cellulose and Halloysite Nanotubes. Sustain. Chem. Pharm..

[3] Zhang D., Ahlivia E.B., Bruce B.B., Zou X., Battino M., Savić D., Katona J., Shen L. (2025). The Road to Re-Use of Spice By-Products: Exploring Their Bioactive Compounds and Significance in Active Packaging. Foods.

[4] Hadidi M., Jafarzadeh S., Forough M., Garavand F., Alizadeh S., Salehabadi A., Khaneghah A.M., Jafari S.M. (2022). Plant Protein-Based Food Packaging Films; Recent Advances in Fabrication, Characterization, and Applications. Trends Food Sci. Technol..

[5] Gupta V., Biswas D., Roy S. (2022). A Comprehensive Review of Biodegradable Polymer-Based Films and Coatings and Their Food Packaging Applications. Materials.

[6] Pavani M., Singh S.K., Singha P. (2024). Chitosan from Agro-Waste for Food Packaging Applications. Agro-Waste Derived Biopolymers and Biocomposites.

[7] Gonçalves L.d.O., Farias P.M.D., Freitas T.F.d., Zago L., Moreira R.F.A., Maniglia B.C., Fai A.E.C. (2025). Babassu-Derived Sachet for *Pereskia aculeata*–Based Oily Sauce: A Bio-Compostable Single-Use Packaging Option. J. Food Sci..

[8] Ferreira D.C.M., Molina G., Pelissari F.M. (2020). Effect of Edible Coating from Cassava Starch and Babassu Flour (*Orbignya phalerata*) on Brazilian Cerrado Fruits Quality. Food Bioproc. Technol..

[9] Galante M., Brassesco M.E., Maragoni Santos C., Beres C., Fai A.E.C., Cabezudo I. (2025). Grape Pomace as a Natural Source of Antimicrobial Agents for Food Preservation. Front. Nutr..

[10] Politi F.A.S., Mello J.C.P.d., Migliato K.F., Nepomuceno A.L.A., Moreira R.R.D., Pietro R.C.L.R. (2011). Antimicrobial, Cytotoxic and Antioxidant Activities and Determination of the Total Tannin Content of Bark Extracts Endopleura Uchi. Int. J. Mol. Sci..

[11] Silva L.R., Teixeira R. (2015). Phenolic Profile and Biological Potential of Endopleura Uchi Extracts. Asian Pac. J. Trop. Med..

[12] Oliveira R.T., dos Santos Rolim C.S., do Nascimento Rolim L., de Sousa Gomes M.L., Martins G.A.S., de Castro L.M., do Nascimento W.M., Saraiva-Bonatto E.C., de Cássia Saraiva Nunomura R., Lamarão C.V. (2021). Endopleura Uchi—A Review about Its Nutritional Compounds, Biological Activities and Production Market. Food Res. Int..

[13] Rosa A., Hoscheid J., Garcia V., de Oliveira Santos Junior O., da Silva C. (2024). Phytochemical Extract from Syzygium Cumini Leaf: Maximization of Compound Extraction, Chemical Characterization, Antidiabetic and Antibacterial Activity, and Cell Viability. Processes.

[14] Ruan Z.P., Zhang L.L., Lin Y.M. (2008). Evaluation of the Antioxidant Activity of *Syzygium cumini* Leaves. Molecules.

[15] Balabram S.K., Tessaro L., Astolfo M.E.d.A., Sponchiado P.A.I., Bogusz Junior S., Maniglia B.C. (2025). Development of NADES–Annatto Seed Extract for Enhancing 3D Printed Food Designed for Dysphagia Patients. Foods.

[16] Bertolo M.R.V., Bogusz Junior S., Mitchell A.E. (2023). Green Strategies for Recovery of Bioactive Phenolic Compounds from Agro-Industrial Wastes (Pomegranate Peels, Almond Hulls, and Elderberry Pomace) Using Natural Deep Eutectic Solvents. ACS Food Sci. Technol..

[17] Bertolo M.R.V., Oliveira L.F.R., Titato G.M., Lanças F.M., Correa D.S. (2025). Sustainable Extraction of Value-Added Compounds from Orange Waste Using Natural Deep Eutectic Solvents. J. Mol. Liq..

[18] Dai Y., van Spronsen J., Witkamp G.-J., Verpoorte R., Choi Y.H. (2013). Natural Deep Eutectic Solvents as New Potential Media for Green Technology. Anal. Chim. Acta.

[19] Airouyuwa J.O., Mostafa H., Ranasinghe M., Maqsood S. (2023). Influence of Physicochemical Properties of Carboxylic Acid-Based Natural Deep Eutectic Solvents (CA-NADES) on Extraction and Stability of Bioactive Compounds from Date (*Phoenix dactylifera* L.) Seeds: An Innovative and Sustainable Extraction Technique. J. Mol. Liq..

[20] Boiteux J., Espino M., Azcarate S., Silva M.F., Gomez F.J.V., Pizzuolo P., de los Angeles Fernandez M. (2023). NADES Blend for Bioactive Coating Design as a Sustainable Strategy for Postharvest Control. Food Chem..

[21] Zhao P., Wang J., Yan X., Cai Z., Fu L., Gu Q., Liu L., Jin H., Fu Y. (2022). Functional Chitosan/Zein Films with Rosa Roxburghii Tratt Leaves Extracts Prepared by Natural Deep Eutectic Solvents. Food Packag. Shelf Life.

[22] Elia Dazat R., Fernandez M.d.l.Á., Espino M., Boiteux J., Silva M.F., Gomez F.J.V. (2024). Biopolymeric Sensor Based on Natural Deep Eutectic Solvents for Monitoring Meat Spoilage. Food Control.

[23] Tadele M.A., Roy V.C., Ho T.C., Chun B.-S. (2025). Extraction of Anthocyanin from Purple Sweet Potato Using Ultrasound-Assisted Natural Deep Eutectic Solvents and Its Application for Smart Packaging Film. Food Bioproc. Technol..

[24] Shivangi S., Dorairaj D., Negi P.S., Shetty N.P. (2021). Development and Characterisation of a Pectin-Based Edible Film That Contains Mulberry Leaf Extract and Its Bio-Active Components. Food Hydrocoll..

[25] Zhu M., Yang P., Zhu L. (2024). Preparation of Modified Atmosphere Packaging Based on the Respiratory Characteristics of Cherry Tomato and Its Freshness Preservation Application. Sci. Hortic..

[26] Álvarez A., Manjarres J.J., Ramírez C., Bolívar G. (2021). Use of an Exopolysaccharide-Based Edible Coating and Lactic Acid Bacteria with Antifungal Activity to Preserve the Postharvest Quality of Cherry Tomato. LWT.

[27] Locali-Pereira A.R., Guazi J.S., Conti-Silva A.C., Nicoletti V.R. (2021). Active Packaging for Postharvest Storage of Cherry Tomatoes: Different Strategies for Application of Microencapsulated Essential Oil. Food Packag. Shelf Life.

[28] Moeini A., Salazar S.A., Gargiulo L., Dougué Kentsop R.A., Mattana M., Genga A., Josi C., Pedram P., Cabrera-Barjas G., Guerra S. (2026). Development of Alginate-Based Active Edible Coating with Brassica Juncea and Raphanus Sativus Sprout Extracts to Extend Tomato Shelf-Life. Food Hydrocoll..

[29] AOAC (2016). Official Methods of Analysis of AOAC International.

[30] Matheus J.R.V., de Freitas T.F., Zago L., Gouvea R., Lima E.R.d.A., Ferreira F.N., de Gois J.S., Luchese C.L., de Andrade C.J., Fai A.E.C. (2025). Olive Leaves Addition on Starch-Pectin Films: Optimization, Characterization, and Evaluation as Edible Hydrosoluble Sachets. Food Bioproc. Technol..

[31] Delgado-González M.J., Carmona-Jiménez Y., Rodríguez-Dodero M.C., García-Moreno M.V. (2018). Color Space Mathematical Modeling Using Microsoft Excel. J. Chem. Educ..

[32] Bertolo M.R.V., Martins V.C.A., Plepis A.M.G., Bogusz S. (2021). Utilization of Pomegranate Peel Waste: Natural Deep Eutectic Solvents as a Green Strategy to Recover Valuable Phenolic Compounds. J. Clean. Prod..

[33] Fernandes C.C., Haghbakhsh R., Marques R., Paiva A., Carlyle L., Duarte A.R.C. (2021). Evaluation of Deep Eutectic Systems as an Alternative to Solvents in Painting Conservation. ACS Sustain. Chem. Eng..

[34] Jessop P.G., Jessop D.A., Fu D., Phan L. (2012). Solvatochromic Parameters for Solvents of Interest in Green Chemistry. Green Chem..

[35] Matheus J.R.V., Maragoni-Santos C., de Freitas T.F., Hackbart E.F.C., Ribeiro-Santos R., Perrone D., de Sousa A.M.F., Luchese C.L., de Andrade C.J., Fai A.E.C. (2024). Starch-Pectin Smart Tag Containing Purple Carrot Peel Anthocyanins as a Potential Indicator of Analogous Meat Freshness. Int. J. Biol. Macromol..

[36] Souza W.F.M., Mariano X.M., Isnard J.L., de Souza G.S., de Souza Gomes A.L., de Carvalho R.J.T., Rocha C.B., Junior C.L.S., Moreira R.F.A. (2019). Evaluation of the Volatile Composition, Toxicological and Antioxidant Potentials of the Essential Oils and Teas of Commercial Chilean Boldo Samples. Food Res. Int..

[37] Velásquez P., Bustos D., Montenegro G., Giordano A. (2021). Ultrasound-Assisted Extraction of Anthocyanins Using Natural Deep Eutectic Solvents and Their Incorporation in Edible Films. Molecules.

[38] Filho L.G.A.d.S., Castro K.N.d.C., Pereira A.M.L., Diniz F.M. (2019). Detecção Da Atividade in Vitro de Compostos Naturais à Base de Plantas: Metodologia Científica.

[39] Hamidi M., Toosi A.M., Javadi B., Asili J., Soheili V., Shakeri A. (2024). In Vitro Antimicrobial and Antibiofilm Screening of Eighteen Iranian Medicinal Plants. BMC Complement. Med. Ther..

[40] Barone A.S., Matheus J.R.V., Luchese C.L., Marques M.R.d.C., de Souza A.M.F., Ferreira W.H., Moreira R.F.A., Fai A.E.C. (2024). Active Antioxidant and Aromatic Films Blended with Persimmon (*Diospyros kaki* L.) and Orange Peel Flour (*Citrus sinensis*) as Sustainable Packaging. J. Vinyl Addit. Technol..

[41] Batu A. (2004). Determination of Acceptable Firmness and Colour Values of Tomatoes. J. Food Eng..

[42] USDA (1976). United States Standards for Grade of Fresh Tomatoes.

[43] Kumar A., Saini C.S. (2021). Edible Composite Bi-Layer Coating Based on Whey Protein Isolate, Xanthan Gum and Clove Oil for Prolonging Shelf Life of Tomatoes. Meas. Food.

[44] AOAC (2005). Official Methods of Analysis: 15th Edition. TrAC Trends Anal. Chem..

[45] Rather J.A., Makroo H.A., Showkat Q.A., Majid D., Dar B.N. (2022). Recovery of Gelatin from Poultry Waste: Characteristics of the Gelatin and Lotus Starch-Based Coating Material and Its Application in Shelf-Life Enhancement of Fresh Cherry Tomato. Food Packag. Shelf Life.

[46] da Silva N., Junqueira V.C.A., Silveira N.F.A., Taniwaki M.H., Santos R.F.S., Gomes R.A.R. (2010). Manual de Métodos de Análise Microbiológica de Alimentos e Água [Food and Water Microbiological Analysis Methods Manual].

[47] Paixão J.A., Tavares Filho E., Bolini H.M.A. (2020). Investigation of Alcohol Factor Influence in Quantitative Descriptive Analysis and in the Time-Intensity Profile of Alcoholic and Non-Alcoholic Commercial Pilsen Beers Samples. Beverages.

[48] Benvenutti L., Sanchez-Camargo A.d.P., Zielinski A.A.F., Ferreira S.R.S. (2020). NADES as Potential Solvents for Anthocyanin and Pectin Extraction from Myrciaria Cauliflora Fruit By-Product: In Silico and Experimental Approaches for Solvent Selection. J. Mol. Liq..

[49] Jurić T., Uka D., Holló B.B., Jović B., Kordić B., Popović B.M. (2021). Comprehensive Physicochemical Evaluation of Choline Chloride-Based Natural Deep Eutectic Solvents. J. Mol. Liq..

[50] Chevé-Kools E., Choi Y.H., Roullier C., Ruprich-Robert G., Grougnet R., Chapeland-Leclerc F., Hollmann F. (2025). Natural Deep Eutectic Solvents (NaDES): Green Solvents for Pharmaceutical Applications and Beyond. Green Chem..

[51] Mohd Fuad F., Mohd Nadzir M., Harun@Kamaruddin A. (2021). Hydrophilic Natural Deep Eutectic Solvent: A Review on Physicochemical Properties and Extractability of Bioactive Compounds. J. Mol. Liq..

[52] Matheus J.R.V., Nogueira T.B.d.B., Pereira A.P.A., Correia T.R., de Sousa A.M.F., Pastore G.M., Pelissari F.M., Miyahira R.F., Fai A.E.C. (2021). Antibacterial Films Made with Persimmon (*Diospyros kaki* L.), Pectin, and Glycerol: An Experimental Design Approach. J. Food Sci..

[53] Guo C., Lv L., Liu Y., Ji M., Zang E., Liu Q., Zhang M., Li M. (2023). Applied Analytical Methods for Detecting Heavy Metals in Medicinal Plants. Crit. Rev. Anal. Chem..

[54] Molina A.K., Corrêa R.C.G., Prieto M.A., Pereira C., Barros L. (2023). Bioactive Natural Pigments’ Extraction, Isolation, and Stability in Food Applications. Molecules.

[55] Rajendran P., Somasundaram P., Dufossé L. (2023). Microbial Pigments: Eco-Friendly Extraction Techniques and Some Industrial Applications. J. Mol. Struct..

[56] Wu H., Zhang G., Zhang Y., Guo P., Wu H., Gao R., Liu T. (2025). Natural Deep Eutectic Solvent (NADES)-Aided Extraction of Bioactive Compounds from Cotton Byproducts for Agricultural Applications: Extraction Optimization, Structural Identification, and Bioactivity Evaluation. Ind. Crops Prod..

[57] Burel C., Kala A., Purevdorj-Gage L. (2021). Impact of PH on Citric Acid Antimicrobial Activity against Gram-negative Bacteria. Lett. Appl. Microbiol..

[58] Yong H., Liu J. (2021). Active Packaging Films and Edible Coatings Based on Polyphenol-rich Propolis Extract: A Review. Compr. Rev. Food Sci. Food Saf..

[59] Yu Y., Li T., Li S., Jia S., Yang X., Cui Y., Ma H., Yan S., Zhang S. (2025). Nature Nano-Barrier: HPMC/MD-Based Lactobacillus Plantarum Pickering Emulsion to Extend Cherry Tomato Shelf Life. Foods.

[60] Chen K., Jiang J., Tian Y., Guo Y., He T., Xie Y., Wu K., Zhu F., Jiang F. (2025). Improved Konjac Glucomannan/Curdlan-Based Emulsion Coating by Mung Bean Protein Addition for Cherry Tomato Preservation. Int. J. Biol. Macromol..

[61] Sganzerla W.G., Pereira Ribeiro C.P., Uliana N.R., Cassetari Rodrigues M.B., da Rosa C.G., Ferrareze J.P., Veeck A.P.d.L., Nunes M.R. (2021). Bioactive and PH-Sensitive Films Based on Carboxymethyl Cellulose and Blackberry (*Morus nigra* L.) Anthocyanin-Rich Extract: A Perspective Coating Material to Improve the Shelf Life of Cherry Tomato (*Solanum lycopersicum* L. var. *cerasiforme*). Biocatal. Agric. Biotechnol..

[62] Li Y., Zhou Y., Wang Z., Cai R., Yue T., Cui L. (2021). Preparation and Characterization of Chitosan–Nano-ZnO Composite Films for Preservation of Cherry Tomatoes. Foods.

[63] Constantino L.V., de Araujo S.R., Suzuki Fukuji A.S., Nogueira A.F., de Lima Filho R.B., Zeffa D.M., Nicio T.T., Oliveira C., Azeredo Gonçalves L.S. (2022). Post-Harvest Characterization and Sensory Analysis of Roma Tomato Cultivars under Organic Cultivation: A Strategy Using Consumers and Chefs. Int. J. Gastron. Food Sci..

[64] Senevirathna S.M.A.A., Jayathunge K.G.L.R., Prasanga G.L.R., Wijesekara W.L.I. (2024). Plant-Derived Composite Edible Coatings for Prolonging the Postharvest Life of Lime (*Citrus aurantiifolia*) and Tomato (*Solanum lycopersicum* L.) under Ambient Storage. ACS Food Sci. Technol..

[65] Marques C., Correia E., Dinis L.-T., Vilela A. (2022). An Overview of Sensory Characterization Techniques: From Classical Descriptive Analysis to the Emergence of Novel Profiling Methods. Foods.

[66] Marwood S., Byrne N., McCarthy O., Heavin C., Barlow P. (2023). Examining the Relationship between Consumers’ Food-Related Actions, Wider Pro-Environmental Behaviours, and Food Waste Frequency: A Case Study of the More Conscious Consumer. Sustainability.

