# Supplementary material for: Babassu Mesocarp-Based Coating with Amazonian Plant Extracts Obtained Using Natural Deep Eutectic Solvents (NADES) for Cherry Tomato Preservation
Source: Foods. 2025 Dec 25;15(1):74. doi: 10.3390/foods15010074 (PMC12786096; doi:10.3390/foods15010074)
Supplement: Supplementary file 1 [file foods-15-00074-s001.zip › foods-4041427-supplementary.pdf]

Supplementary material

BABASSU MESOCARP-BASED COATING WITH AMAZONIAN PLANT EXTRACTS OBTAINED USING NATURAL DEEP EUTECTIC SOLVENTS (NADES) FOR TOMATO PRESERVATION

Carollyne Maragoni-Santos[1]; Camila Marcolongo Gomes Cortat[2]; Lilia Zago[2]; Stanislau Bogusz Junior[3]; Tatiana Castro Abreu Pinto[4]; Jefferson Santos de Gois[5]; Bianca Chieregato Maniglia[3]; Ana Elizabeth Cavalcante Fai[1,2*]

[1]Food and Nutrition Graduate Program, Federal University of the State Rio de Janeiro (UNIRIO), Rio de Janeiro/RJ, Brazil,

[2]Laboratory of Multidisciplinary Practices for Sustainability (LAMPS), Department of Basic and Experimental Nutrition, Institute of Nutrition, Rio de Janeiro State University (UERJ)

[3]São Carlos Institute of Chemistry, University of Sao Paulo, SP, Brazil

[4]Paulo de Goes Institute of Microbiology, Federal University of Rio de Janeiro (UFRJ), Rio de Janeiro/RJ, Brazil

[5]Department of Analytical Chemistry, Rio de Janeiro State University (UERJ), Rio de Janeiro, RJ, Brazil

*Correspondence: ana.fai@uerj.br

Methods

Centesimal Composition

Moisture and ash were determined using the gravimetric method. The lipid content was determined the Soxhlet method. The protein content was determined using the Kjeldahl method and the results are expressed as a percentage of protein using the conversion factor of 6.25. The available carbohydrate content was calculated by difference, which was calculated by subtracting the percentage of moisture, ash, lipids, and proteins from the total composition of the sample. Each sample was measured in triplicate (n = 3) according to the official AOAC methods (AOAC, 2016) and expressed in g 100 g^{-1} dry matter.

Multielement Determination

The multielement profile of the powders of yellow uxi bark and the leaves of Jambolan was recorded using ICP OES (inductively coupled plasma optical emission spectroscopy), model iCAP 6000 (Thermo Scientific, Waltham, MA, USA). Argon with a purity of 99.95% (Air Liquide, Brazil) was used as main, auxiliary and nebulizer gas. The operational parameters for all measurements were: plasma frequency power of 1150 W, a pump speed of 50 rpm, a pump stabilization time of 2 s, an auxiliary gas flow rate of 1 L min^{-1}, and a radial plasma view. The samples were digested in a microwave reaction system (model Multiwave PRO, Anton Paar, Graz, Austria) equipped with 24 Teflon® flasks with an internal volume of 50 mL, using HNO_3 65% v/v (Quimis, Brazil) and H_2O_2 35% w/v (NEON, Brazil) at a power of 1200 W. Heating was performed with a heating program up to 200 °C for 10 min with a break of 15 min and cooling to 70 °C (22 min). The wavelengths monitored were: Aluminum (Al) – 308.215 nm; Barium (Ba) – 455.4 nm; Calcium (Ca) – 422.673 nm; Cadmium (Cd) – 228.802 nm; Cobalt (Co) – 228.616 nm; Chromium (Cr) – 283.563 nm; Copper (Cu) – 324.754; Iron (Fe) – 238.204 nm; Potassium (K) – 769.886 nm; Magnesium (Mg) – 279.553 nm; Manganese (Mn) – 257.610 nm; Sodium (Na) – 589.592 nm; Phosphorus (P) – 214.914 nm; Lead (Pb) – 220.353 nm; Sulfur (S) – 182.034 nm; Selenium (Se) – 196.090 nm; Strontium (Sr) – 407.771 nm; Zinc (Zn) – 213.856 nm. Single element solutions (Specsol, Jacareí, São Paulo, Brazil) were used to prepare calibration curves (which ranged from 0.01 mg L^{-1} to 10 mg L^{-1} according to the analyte), according to dilutions required. All determinations were performed in triplicate (Matheus et al, 2025).

Color parameters

The powders of yellow uxi bark and the leaves of Jambolan were evaluated in triplicate for color parameters (L^*, a^*, b^*) using a colorimeter (3nh, Colorimeter Spectrometer Y53020, China) configured with a 10° angle and illuminant D_{65}. The resulting color parameters were plotted using Microsoft Excel in the color charts proposed by Delgado-González et al- https://doi.org/10.1021/acs.jchemed.7b00681. The chroma value (C_{ab}*) was calculated considering Eq. S1 and and the color difference (ΔE^*) was calculated according to Eq. S2

$$C*_{ab=\sqrt{a^{*2}+b^{*2}}} \quad \text{(S1)}$$

$$\Delta E = \sqrt{(L*-L*_0)^2 + (a*-a*_0)^2 + (b*-b*_0)}^2 \quad \text{(S2)}$$

Results

Table S1. Characterization of Uxi bark powder and Jambolan leaves powder

	Uxi bark	Jambolan leaves
Proximate composition		
Moisture (g 100 g^{-1})	7.24 ± 0.17	8.19 ± 0.04
Ash (g 100 g^{-1})	4.08 ± 0.07	5.51 ± 0.01
Protein (g 100 g^{-1})	5.08 ± 0.37	6.47 ± 0.71
Lipids (g 100 g^{-1})	2.01 ± 0.58	1.98 ± 0.72
Carbohydrate (g 100 g^{-1})	81.58 ± 0.45	77.85 ± 0.61
Multielement composition		
Aluminum (Al, μg g^{-1})	760 ± 12	243 ± 6
Barium (Ba, μg g^{-1})	2.5 ± 0.1	61 ± 1
Calcium (Ca, mg g^{-1})	7.16 ± 0.082	9.78 ± 0.265
Copper (Cu, μg g^{-1})	16 ± 1	7.6 ± 2
Iron (Fe, μg g^{-1})	394 ± 13	172 ± 3
Potassium (K, mg g^{-1})	2.32 ± 0.019	8.864 ± 0.167
Magnesium (Mg, μg g^{-1})	521 ± 5	3453 ± 96
Manganese (Mn, μg g^{-1})	24 ± 0	97 ± 2
Sodium (Na, μg g^{-1})	333 ± 7	<LOD
Phosphorus (P, μg g^{-1})	107 ± 12	847 ± 29
Sulfur (S, μg g^{-1})	817 ± 75	1024 ± 119
Strontium (Sr, μg g^{-1})	69 ± 1	55 ± 1
Zinc (Zn, μg g^{-1})	Present	24 ± 1
Color parameters		
L	21.77 ± 1.22	32.15 ± 2.09
a*	12.11 ± 0.43	5.61 ± 0.16
b*	16.1 ± 0.6	17.09 ± 0.62
Chroma value	20.14 ± 0.74	17.99 ± 0.63
ΔE	12.3 ± 1.02	

[a] <LOD in Uxi bark powder and Jambolan leaves powder for elements: Cadmium (3 μg g–1); Cobalt (3 μg g–1); Chromium (2 μg g–1); Lead (21 μg g–1) Selenium (78 μg g–1); [b]Present- Zinc in yellow uxi bark powder is between the LOD (3 μg g^{-1}) and the LOQ (9 μg g^{-1}). [c]Abbreviations: LOD = limit of detection; LOQ = limit of quantification.

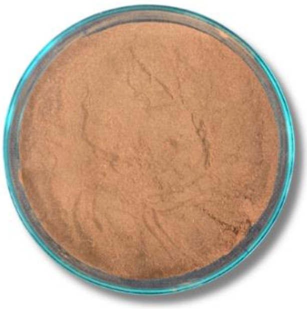

Uxi bark powder

(A)

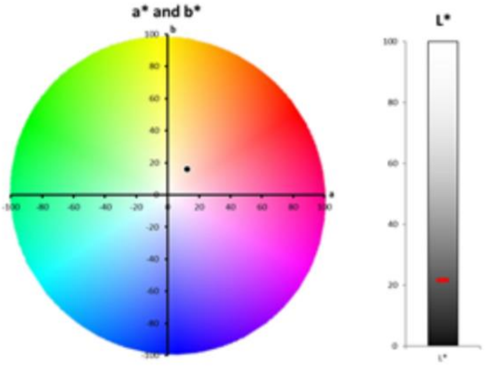

Color parameters of the Uxi bark powder

(B)

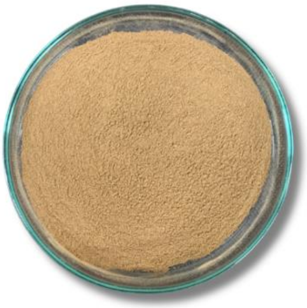

Jambolan leaves power

(C)

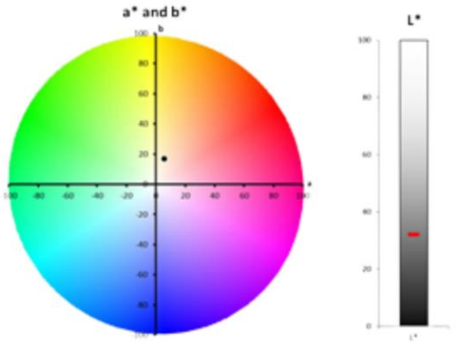

Color parameters of the Jambolan leaves powder

(D)

Figure S1. (A) Uxi bark powder <100 mesh; (B) Color parameters of the uxi bark; (C) Jambolan leaves powder <100 mesh; and (D) Jambolan leave powders

Table S2. Questionnaire questions about cherry tomato consumption habits and perceptions of waste

**Do you usually consume cherry tomatoes?**
**For what reasons do you consume or avoid consuming cherry tomatoes?**
**How often do you throw away cherry tomatoes because they are spoiled?**
**In your opinion, which of these aspects is cherry tomato waste associated with?**
**What do you usually do when you notice that your cherry tomatoes are starting to spoil?**

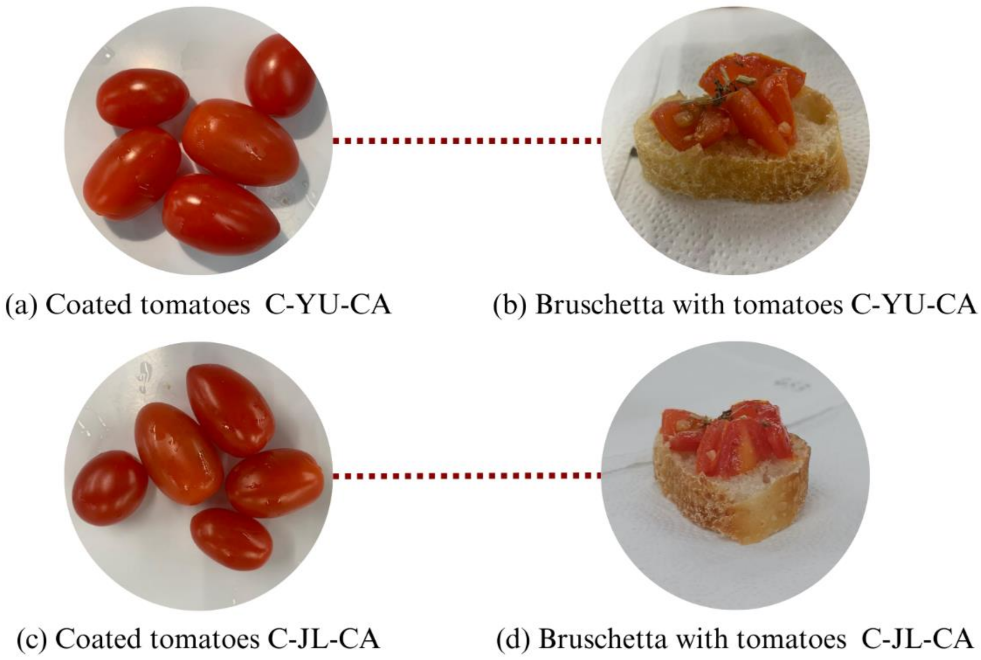

Figure S2. Coated tomatoes with (a) NADES-Uxi extract (C-YU-CA) and (c) NADES-Jambolan leaf extract (C-JL-CA), and their respective bruschettas (b) and (d).

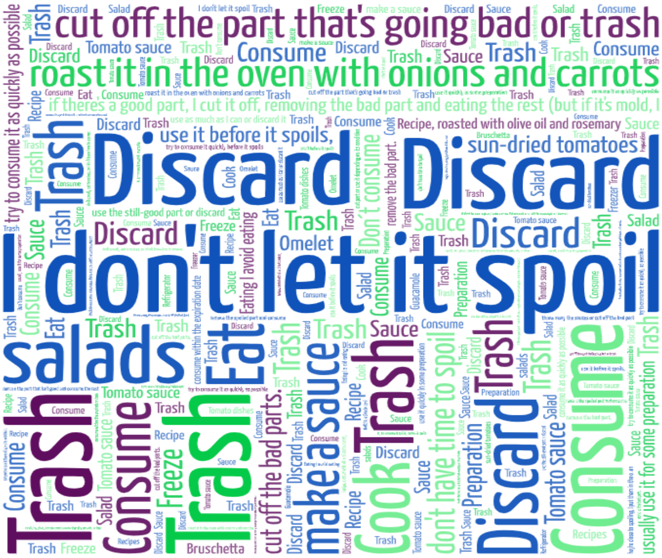

Figure S3. Word cloud of tasters' comments (Answer to the question: “What do you usually do when you notice that your cherry tomatoes are starting to spoil?”).
